# A machine learning tool for early identification of celiac disease autoimmunity

**DOI:** 10.1038/s41598-024-80817-0

**Published:** 2024-12-28

**Authors:** Michael Dreyfuss, Benjamin Getz, Benjamin Lebwohl, Or Ramni, Daniel Underberger, Tahel Ilan Ber, Shlomit Steinberg-Koch, Yonatan Jenudi, Sivan Gazit, Tal Patalon, Gabriel Chodick, Yehuda Shoenfeld, Amir Ben-Tov

**Affiliations:** 1 Predicta Med Analytics Ltd., Ramat Gan, Israel; 2 Celiac Disease Center, Department of Medicine, Columbia University Irving Medical Center, New York, NY USA; 3 Kahn Sagol Maccabi Research & Innovation Center, Maccabi Healthcare Services, Tel Aviv, Israel; 4 Zabludowicz Center for Autoimmune Diseases, Chaim Sheba Medical Center, Tel Hashomer, Israel; 5 Faculty of Medicine, Tel Aviv University, Tel Aviv, Israel; 6 PhaseV, Tel Aviv, Israel

**Keywords:** Gastrointestinal diseases, Translational research, Preclinical research, Information technology, Risk factors

## Abstract

Identifying which patients should undergo serologic screening for celiac disease (CD) may help diagnose patients who otherwise often experience diagnostic delays or remain undiagnosed. Using anonymized outpatient data from the electronic medical records of Maccabi Healthcare Services, we developed and evaluated five machine learning models to classify patients as at-risk for CD autoimmunity prior to first documented diagnosis or positive serum tissue transglutaminase (tTG-IgA). A train set of highly seropositive (tTG-IgA > 10X ULN) cases (*n* = 677) with likely CD and controls (*n* = 176,293) with no evidence of CD autoimmunity was used for model development. Input features included demographic information and commonly available laboratory results. The models were then evaluated for discriminative ability as measured by AUC on a distinct set of highly seropositive cases (*n* = 153) and controls (*n* = 41,087). The highest performing model was XGBoost (AUC = 0.86), followed by logistic regression (AUC = 0.85), random forest (AUC = 0.83), multilayer perceptron (AUC = 0.80) and decision tree (AUC = 0.77). Contributing features for the XGBoost model for classifying a patient as at-risk for undiagnosed CD autoimmunity included signs of anemia, transaminitis and decreased high-density lipoprotein. This model’s ability to distinguish cases of incident CD autoimmunity from controls shows promise as a potential clinical tool to identify patients with increased risk of having undiagnosed celiac disease in the community, for serologic screening.

**Supplementary Information:**

The online version contains supplementary material available at 10.1038/s41598-024-80817-0.

## Introduction

Celiac disease (CD) is an immune-mediated disease characterized by small bowel enteropathy triggered by dietary exposure to gluten. The classic clinical presentation of CD includes signs of malabsorption and gastrointestinal symptoms such as abdominal pain, bloating and diarrhea^1,2^. However, many patients experience predominantly non-specific extra-intestinal symptomatology^3,4^, or are asymptomatic^5^. The diverse manifestations of CD can make it challenging for primary care providers (PCPs) to identify and diagnose CD in the general population^6^. Indeed, a majority of adult patients with CD today are likely undiagnosed^5–8^. Patients who eventually receive a diagnosis experience a mean delay of eleven years from symptom onset to diagnosis, with more than half reporting a delay of five or more years till the diagnosis is established^6^. CD has an estimated global prevalence of over 1%^9^, and a rising incidence in recent years^6,10–12^. Diagnostic delays and underdiagnosis of CD therefore represent an important healthcare problem^13^.

Screening for CD is typically performed by highly sensitive and widely available serum tests for CD autoimmunity (CDA): the most accurate and commonly used being the assay for antibodies to tissue-transglutaminase (tTG-IgA)^14^. Seropositivity is suggestive of underlying CD, and such patients typically undergo endoscopic evaluation to establish or rule out the diagnosis of CD via biopsy of intestinal mucosa^15^. High seropositivity (tTG-IgA > 10X ULN) is associated with a high (> 95%) positive predictive value (PPV) for villous atrophy and, in the proper clinical setting, is considered sufficient for diagnosis without a biopsy in children and possibly in adults^7,14–16^.

Clinical guidelines do not provide clear and consistent definitions on when to screen for CDA. General population screening is not currently recommended^17^, although screening high-risk patients may be warranted^18,19^. What defines high-risk groups for CD varies somewhat between reports, although the focus has typically been on a family history of CD and medical comorbidities with established associations with CD^20^. Beyond these factors, laboratory abnormalities such as iron-deficiency anemia are common among patients with CD^21^. While PCPs may be aware of specific risk-factors, signs and symptoms of undiagnosed CD, more subtle combinations of clinical features may go missed.

Machine learning (ML) algorithms have the potential to use existing data within a patient’s electronic medical record (EMR) to provide risk assessments to providers^22^. ML models have been developed to alert intensive care unit physicians to patients at risk of circulatory failure^23^, to identify clinically significant portal hypertension in non-alcoholic steatohepatitis patients from pathology reports^24^, to predict incident hypertension^25^ and hypertension outcomes^26^, to identify patients with undiagnosed psoriatic arthritis^27^, hepatitis C^28^, to predict dementia onset^29^, IgA nephropathy^30^, future Parkinson’s disease diagnosis^31^, and to flag patients at risk of advanced colorectal cancer^32^. One previous study that attempted to develop ML models to identify patients with incident CD using a variety of modeling methods found that the models were not consistently better than chance^33^. Another study showed positive results, but the study size was small and the models relied on symptoms extracted from unstructured clinical documents and diagnostic codes^34^. Neither study included objective laboratory test results as predictive input features, which may have hindered performance and limited generalizability.

The goal of the current study was to develop and assess a prescreening EMR-based tool to classify adult and adolescent patients by risk of having unidentified CD autoimmunity using commonly available clinical features. Five algorithms were trained and tested: logistic regression, decision tree, random forest, XGBoost and multilayer perceptron. Each algorithm was then assessed on discriminative ability as measured by estimated area under the ROC (AUC). Input features included age, biological sex and results from commonly available laboratory tests performed as part of complete blood counts and comprehensive metabolic, iron and lipid panels. Incident cases were identified from a large retrospective community-based dataset using results from tTG-IgA testing. Highly seropositive cases (tTG-IgA > 10X ULN) with probable underlying CD were used for model training and evaluation against cohorts of controls with no evidence of disease. Performance was additionally assessed for the highest performing model in a test set consisting of a cohort of seropositive cases (tTG-IgA > 2X ULN) who may require endoscopic evaluation for CD and a cohort of controls. In both test sets AUC was assessed at multiple time points before first documented evidence of CD autoimmunity.

## Methods

### Dataset

The dataset for this retrospective study consisted of deidentified EMR data from Maccabi Health Services (MHS), Israel’s 2nd largest health maintenance organization (HMO)^35^. Data were accessed via the Kahn Sagol Maccabi Research and Innovation Centre (KSM), and extracted using the MDClone platform (version 5.5.0.4; https://www.mdclone.com/*)*, a proprietary software. The dataset was de-identified by KSM, and no personal identifying information was made available to the researchers. The dataset contains records from 2,963,864 unique patients with longitudinal data, as members rarely change HMOs. Unstructured data, including progress notes and pathology or procedure reports were not made available by KSM. The study therefore relied entirely on the structured data, specifically patient demographics and laboratory results. Approval for use of the dataset and the retrospective analysis was obtained by the Maccabi institutional review board (approval #0052-20-MHS), and the study was conducted in accordance with the Declaration of Helsinki and all relevant guidelines and regulations. Informed consent was waived as all identifying information had been removed by KSM.

### Study cohort definitions

Patients were eligible for inclusion if they (1) were MHS members during the study period (2005–2021), and (2) joined MHS before 2005. The eligible population was randomly split into train (80%) and test (20%) sets. The split was performed by assigning patients with digits 2 or 6 as the third to last digit in their randomly generated hash ID to test, and all others to train.

In this study we distinguish between seropositive cases in general and highly seropositive cases with likely underlying CD. Cohorts of highly seropositive cases were selected in both train and test sets, and an additional cohort of seropositive cases was identified using case identification criteria (CIC) described below.

Highly seropositive cases were defined as patients having at least one documented tTG-IgA test ≥ 10X ULN, which has an extremely high (> 95%) PPV for duodenal biopsy proven CD^7,36–38^. CIC for seropositive cases included all patients with at least one documented tTG-IgA > 2X ULN. This definition is more sensitive for underlying CD, but also includes a higher proportion of cases that would not have pathologic evidence of disease^7^. Reports of procedures including endoscopic evaluations were not available to researchers, so pathologic evidence of CD could not be confirmed or ruled out.

To reduce the possibility that cases were not newly diagnosed, cases were excluded if they had a history of tTG-IgA levels within normal limits before their first positive tTG-IgA, or a CD diagnosis code > 1 year before their first positive tTG-IgA. Providers may order serology on patients with known CD to check patient compliance with a gluten-free diet (GFD), so such cases may have been previously diagnosed and therefore not incident cases relevant for this study. For each seropositive case, the earlier of the first positive serology or first diagnosis of CD was defined as that patient’s index date.

Screening serology was performed with one of two commercial kits used during the study period: Celiakey (Thermo Fisher Scientific-USA) for years 2005–2011 and Elia (Thermo Fisher Scientific-USA) for years 2012–2021, each with manufacturer established ULN values of > 5 U/mL, and > 7 U/mL respectively.

Controls were identified as eligible MHS patients with no documented CD diagnosis code and no serologic evidence of CD autoimmunity. One cohort of controls was selected to match the train set cases and two cohorts of controls in the test set were selected to match the two cohorts of cases (highly seropositive and seropositive). Controls were matched to cases by years of data availability at the maximal possible ratio of controls to cases. Controls were assigned the same index dates as the case to which they were matched. Controls were not matched by demographic characteristics to allow the model to learn the relationship between these features for predicting incident CD seropositivity.

**Fig. 1:**
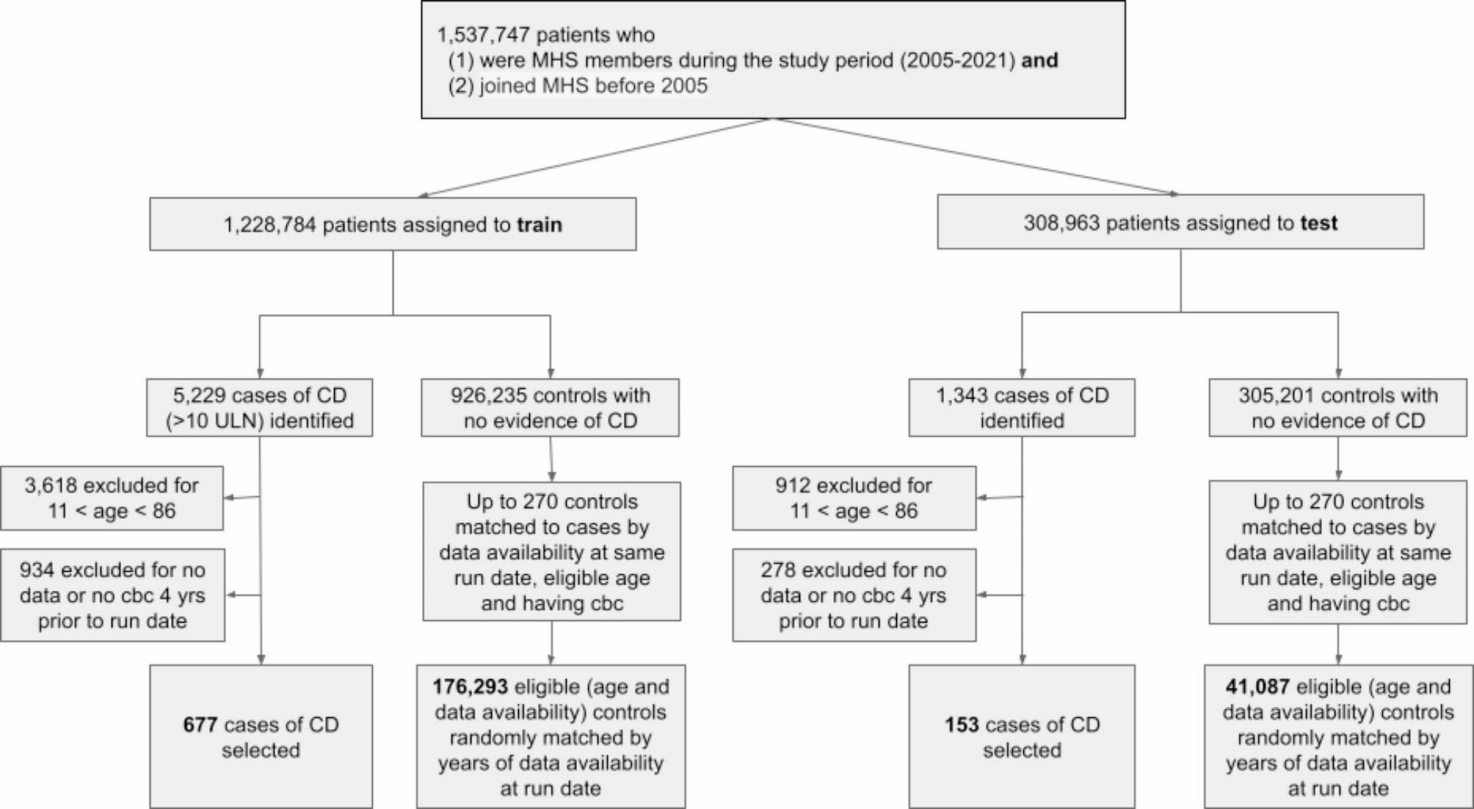
Flow chart of patient selection. Highly seropositive (tTG-IgA > 10X ULN) cases with no previous evidence of celiac disease seropositivity were identified and excluded if at their index date they were (i) age < 12 or age > 85 years-old, (ii) members of MHS for fewer than four years, and (iii) had no documented complete blood count (CBC) during the four years prior to their index date. Controls were matched to cases by years of data availability at the assigned run date where they met eligibility criteria.

Each patient included in the train or test sets was additionally assigned a run date, defined as the first of July of the calendar year preceding the patient’s index date. This gap between run date and index date is referred to as the one-year gap, referring to the mean time between run date and index dates of the cohort. The gap between the run date and the index date was added to account for potential clinical suspicion for CD immediately preceding the index date. Analyses were also conducted at further time gaps of up to four years prior to index dates to test the ability of the model to identify patients years before initial suspicion of disease. This methodology was described in detail in a previous report on patients with psoriatic arthritis^27^. Patients were additionally excluded if on their run date they did not meet the following criteria: (i) age ≥ 12 and age ≤ 85 years-old, (ii) members of MHS for at least four years, and (iii) at least one complete blood count (CBC) during the four years prior to their index date. The patient selection process is depicted in Fig. 1.

### Model development

Five candidate models were developed to classify patients as at-risk or not for having undiagnosed CD autoimmunity. Each model was fit to the training data using the following algorithms: logistic regression, decision tree, random forest, XGBoost and multilayer perceptron. Logistic regression estimates the log-odds of an outcome by linear combination of weighted input features. The estimate can then be converted into a probability using a logit function, which can be used for binary classification given a predefined cutoff. A decision tree performs classification by recursively partitioning based on the given set of input features. Random forest is a method that uses multiple decision trees, and then performs classification based on the result of the majority of the decision trees^39,40^. XGBoost builds multiple decision trees sequentially using gradient boosting to minimize the errors made by previous trees^26,39–41^. A multilayer perceptron is a type of artificial neural network that consists of an input layer, one or multiple hidden layers and an output layer^40^. Neurons of the input layer represent input features, which activate neurons in the hidden layer to produce an output from the output layer. This output can then be converted into a classification. The models were trained and evaluated using data available during the three years prior to the run date. Training was performed at a one-year time gap between run dates and index dates. All models were implemented using the scikit-learn library v1.3^42^ and the scikit-learn compatible XGBoost library.

Candidate independent predictors consisted of basic demographic information (biological sex and age at run date), and laboratory test results extracted from the structured data (Supplementary Table 1). Laboratories included as features all individual components of the comprehensive metabolic panel and complete blood count with differential, ferritin and high-density lipoprotein (HDL) as patients with celiac often have iron deficiency anemia^4^ and low HDL^43^. The most recent available laboratory result of each type log-transformed by sex and age group. Missing data were given a special indication and the model treated these as null values. For the logistic regression, null values were replaced by median values for the feature as calculated by biological sex and age group. SHapley Additive exPlanations (SHAPs) were used both for model selection and to explain the output of the XGBoost model^44^.

Models were developed to provide each patient with an output score given the patient’s input features. The score can be converted at a given threshold to perform binary classification of positive (at-risk for undiagnosed CD) or negative (not at-risk for undiagnosed CD) classes. The predicted classes assigned to each patient are then evaluated against the ground truth labels established by the CIC as described above (i.e. incident case of CD autoimmunity or control with no documented evidence of CD).

The hyperparameters for each algorithm were selected by performing five-fold cross validation within the train set and optimizing for average precision (Supplementary Table 2). Each model was then retrained on the entire train set with the selected hyperparameters to produce one model with each of the five algorithms for evaluation on the test set.

### Model selection and evaluation

To test the ability of the models to identify incident CD seropositivity prior to the first documented evidence of disease, the performance of each model was assessed one year prior to patients’ run dates in the test set with cohorts of highly seropositive cases and controls. The model with the highest AUC was then additionally evaluated at run dates of two-, three- and four-year time gaps prior to each patient’s run date, as well as at all four time gaps in the test set consisting of seropositive patients and controls. Tests at each time gap were performed on the same base cohort selected according to the criteria described above. Patients from each test cohort who did not meet eligibility criteria at previous time gaps were removed from analyses.

## Results

Cohorts of cases of CDA and controls in the train and test sets are described in Table 1.

**Table 1:** Descriptive characteristics of the train and test cohorts. The first test cohort includes cases of celiac disease autoimmunity with high seropositivity (tTG-IgA > 10X ULN) and matched controls. The second test cohort includes all cases of seropositivity with tTG-IgA > 2X ULN.

	Patients, *n*	Age, mean (SD), y	Female sex, *n* (%)
Train cohort			
Controls	176,293	50.7 (18.2)	110,978 (63.0%)
Highly seropositive cases	677	37.8 (17.1)	499 (73.7%)
Highly seropositive CD autoimmunity			
Controls	41,087	49.9 (17.5)	25,657 (62.4%)
Highly seropositive cases	153	36.2 (16.5)	114 (74.5%)
Seropositive CD autoimmunity			
Controls	78,923	49.9 (17.5)	49,483 (62.8%)
Seropositive cases	301	37.1 (17.1)	210 (69.8%)

**Fig. 2:**
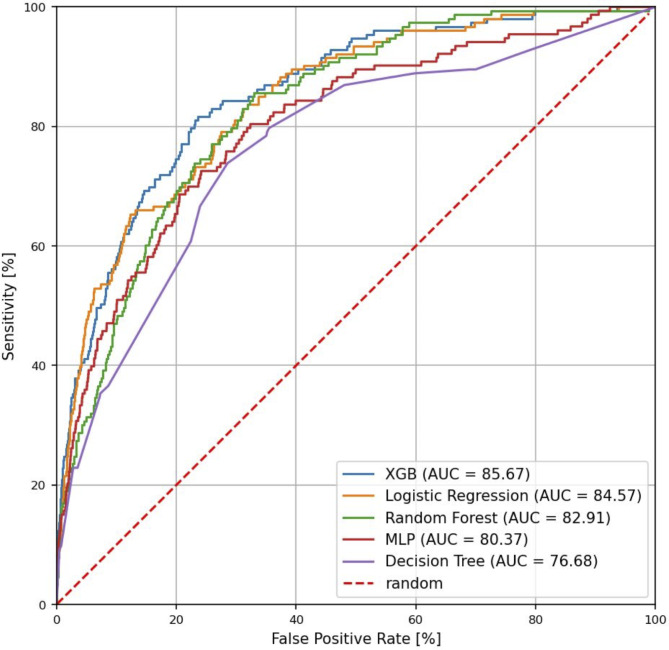
Receiver operating characteristics (ROC) plot for identification of patients with highly seropositive (tTG-IgA > 10X ULN) celiac disease autoimmunity versus controls one year prior to first documented evidence of disease. The performance of five modeling modalities is compared: XGBoost (XGB), logistic regression, random forest, multilayer perceptron (MLP) and decision tree. Figure prepared with Matplotlip v3.8 (https://matplotlib.org/).

**Fig. 3:**
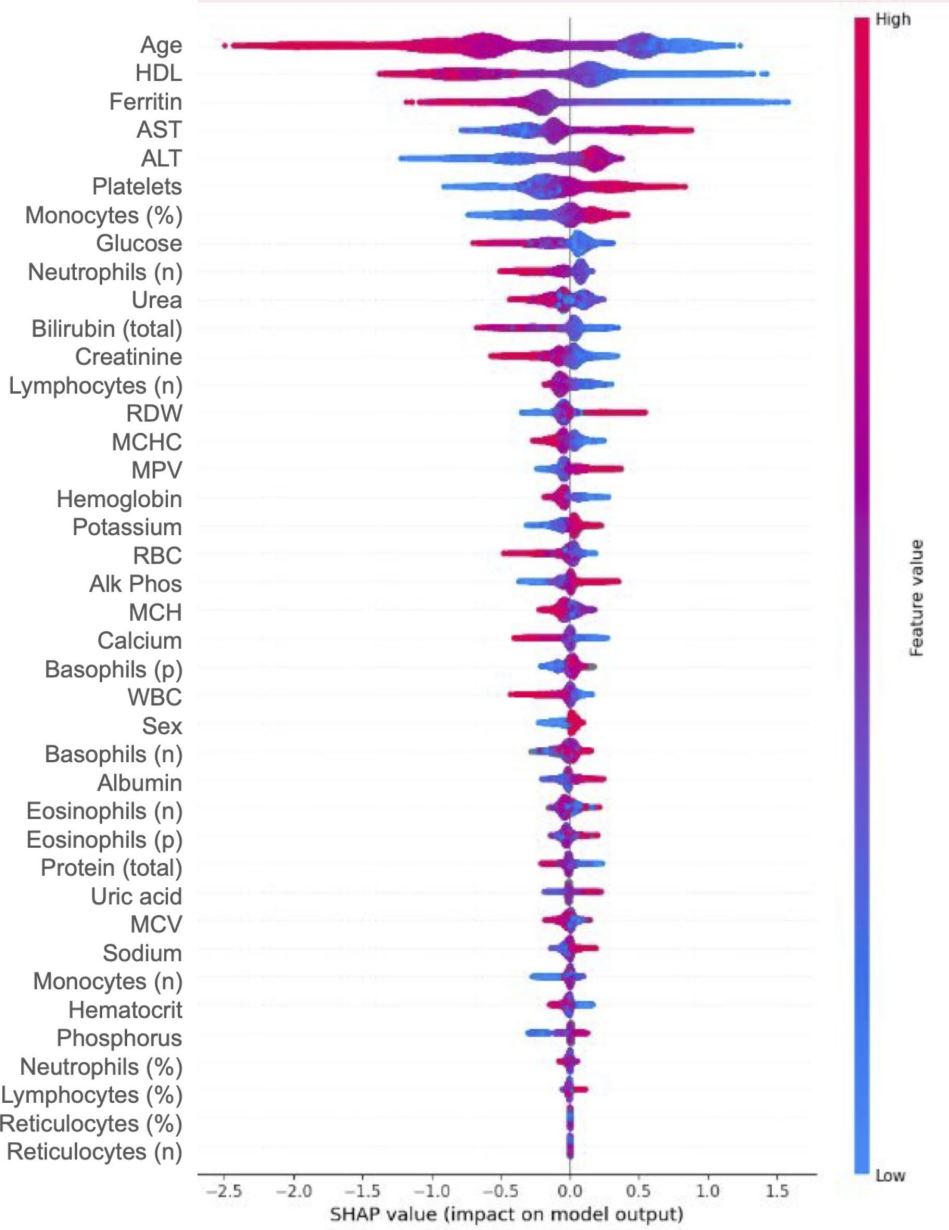
SHAP plot depicting contribution of the input features at the one year time gap prior to first documentation of celiac disease (CD) autoimmunity for the XGBoost model. Positive SHAP values contribute positively to classification of a patient as at-risk for undiagnosed CD autoimmunity. A higher value for a given feature is indicated in red, with lower values in blue. Biological sex was coded as a binary: 1 = Female; 0 = Male. Figure prepared with SHAP v0.42 (https://shap.readthedocs.io).

Performance for each model on the high seropositivity test cohort at a gap of one year are shown in Fig. 2. At the one-year gap, discriminatory AUC was highest for the XGBoost model at 0.86, followed by the models using logistic regression (AUC = 0.85), random forest (AUC = 0.83), multilayer perceptron (AUC = 0.80) and decision tree (AUC = 0.77). Feature contributions for the XGBoost model are depicted for this test by SHAP analysis (Fig. 3). The XGBoost also had the best performance *a*t the two-, three-, and four-year gaps, with AUC values of 0.83, 0.82 and 0.81 respectively (Supplementary Table 3).

The models was then tested on the test set consisting of seropositive cases and controls. The XGBoost model’s ability to distinguish between cases and controls in this test set was assessed by AUC as 0.79, 0.77, 0.75, 0.75 at gaps of one, two, three and four years (Supplementary Table 4).

## Discussion

In the current study, we describe the development and comparative evaluation of five ML models for identifying adult and adolescent patients with incident CD autoimmunity prior to the first documented evidence of disease. Based on AUC, XGBoost exhibited the strongest ability of the models to distinguish between cases of highly seropositive cases of CD autoimmunity and controls one year prior to initial documentation, followed by the models using logistic regression, random forest, multilayer perceptron and decision tree respectively. XGBoost is a particularly robust ML method for tabular data, which often produces the best performance compared to other ML methods^26,30,39–41^. This model showed excellent discriminatory ability (AUC > 0.80) between cases of highly seropositive patients with likely CD at gaps of one, two, three and four years. The model also showed good discriminatory ability (AUC > 0.7) at all time gaps for more broadly defined seropositive cases compared to controls. These findings suggest the potential utility of this model as a prescreening tool to identify patients at risk of having CDA for eventual evaluation for CD. The final model achieved these results using commonly available laboratory results and demographic features.

The relationships between demographic and lab features of the XGBoost model as depicted in the SHAP are consistent with established phenomena among patients with untreated CD. The model identified decreased hemoglobin, ferritin, mean cell hemoglobin (MCH), mean cell hemoglobin concentration (MCHC) and mean cell volume (MCV) as predictive of undiagnosed CD autoimmunity. These laboratory findings are indeed characteristic of anemia secondary to malabsorption of dietary iron and chronic inflammation in the setting of CD^1^. Increased liver function tests (LFTs), specifically alanine transaminase (ALT) and aspartate transferase (AST) and alkaline phosphatase contributed positively to classification as at-risk for the model. In patients with newly diagnosed CD, 20–50% of patients have elevated LFTs, and undetected CD accounts for an estimated 4% of cases of unexplained transaminitis^45,46^. Low high density lipoprotein (HDL) has frequently been associated with untreated CD^47^, and the combination of unexplained iron-deficiency anemia and low HDL may be particularly suggestive of CD^48^. For the model, low HDL contributed to a positive classification as at-risk for undiagnosed CD autoimmunity. Longitudinal studies have found that these lab abnormalities typically resolve after initiation of a GFD, including anemia^49^, transaminitis^50^, and low HDL^51,52^, further highlighting the importance of identifying undiagnosed cases of CD early.

Earlier identification and treatment of CD have been shown to have clinical benefits: screen-detected asymptomatic and mildly symptomatic adult patients typically show improved intestinal histology and reduced serum autoantibody levels after following adherence to a GFD^53,54^. Late diagnosis in contrast has been associated with unfavorable clinical outcomes. Patients diagnosed after 40 years of age are more likely than younger patients to show persistent signs of intestinal mucosal injury, including villous blunting and intraepithelial lymphocytes in the duodenum despite following a strict GFD^55^. Among symptomatic patients, diagnostic delays are associated with poorer long-term outcomes even after initiation of a GFD, including persistent gastrointestinal and extra-gastrointestinal symptoms^3,4,56^, increased utilization of healthcare resources, and lower reported quality of life measures^8,57,58^. Nevertheless CD is a highly heterogeneous disease, and the long-term benefits of early identification by screening should be established in future studies^59^.

To our knowledge, this is the first report of a ML model showing the ability to identify cases of incident CDA from controls within a large community-based setting using only commonly available laboratory results, biological sex and age in adults and adolescents. This tool may have clinical value as a prescreening tool to identify patients who should be evaluated for CDA. Patients who are found to be seropositive can then undergo further evaluation for CD according to clinical guidelines, including additional serologic testing or endoscopic evaluation with multiple biopsies^60^.

A major strength of the study was the size and continuity of follow-up in the longitudinal data set, which allowed for large patient cohorts with rich historical data. An additional strength is that by using commonly available lab results and demographic information, the model can be portably implemented in most EMR systems. Follow-up studies are needed to evaluate the robustness of the models to other datasets with different population demographics and EMR systems. Additionally, prospective studies should explore the PPV that a flagged patient is seropositive for CDA, and for undiagnosed CD. This model, if validated, may assist PCPs in identifying patients with CDA at point of care, or health systems identifying patients in their populations who may benefit from screening for CDA.

The study also had limitations. Due to lack of access to unstructured data such as clinical documentation, patients were selected by the proxy of test results rather than by endoscopy results. Some patients selected as cases with CDA may not have had CD. Diagnostic status as reported in clinical documents such as were not made available to establish CD status. Highly seropositive patients are very likely to have CD^7^. Some controls may also have CD that was not documented or undiagnosed at the time the study was conducted. Additionally, there may be heterogeneity in the full population that was not captured in the cohorts of cases and controls used for model training and evaluation. Future studies should examine the robustness of the model, and its ability to perform on different populations and health care systems that may differ from MHS through retrospective and prospective validation studies.

In conclusion, this study presents a ML model based on a large population dataset that can identify adults and adolescents at risk of undiagnosed CD autoimmunity using commonly available structured clinical and demographic data.

## Electronic supplementary material

Below is the link to the electronic supplementary material.

Supplementary Material 1

## Data Availability

The authors do not have permission to share the data itself, which can be accessed through agreement with KSM. Statistics and analytics can be provided upon request from Michael Dreyufss or Benjamin Getz.

## References

[1] Reilly, N. R., Fasano, A. & Green, P. H. R. Presentation of celiac disease. *Gastrointest. Endosc. Clin. N. Am.* **22**, 613–621 (2012). 23083982 10.1016/j.giec.2012.07.008

[2] Catassi, C., Verdu, E. F., Bai, J. C. & Lionetti, E. Coeliac disease. *Lancet* **399** (10344), 2413–2426 (2022). 35691302 10.1016/S0140-6736(22)00794-2

[3] Laurikka, P., Nurminen, S., Kivelä, L. & Kurppa, K. Extraintestinal manifestations of celiac disease: early detection for better long-term outcomes. *Nutrients* **10**, 1015 (2018). 30081502 10.3390/nu10081015 PMC6115849

[4] Jericho, H., Sansotta, N. & Guandalini, S. Extraintestinal manifestations of celiac disease: effectiveness of the gluten-free diet. *J. Pediatr. Gastroenterol. Nutr.* **65**, 75–79 (2017). 28644353 10.1097/MPG.0000000000001420

[5] Choung, R. S. et al. Prevalence and morbidity of undiagnosed celiac disease from a community-based study. *Gastroenterology* **152**, 830–839e5 (2017). 27916669 10.1053/j.gastro.2016.11.043 PMC5337129

[6] Lebwohl, B. & Rubio-Tapia, A. Epidemiology, presentation, and diagnosis of celiac disease. *Gastroenterology* **160**, 63–75 (2021). 32950520 10.1053/j.gastro.2020.06.098

[7] Kvamme, J. M., Sørbye, S., Florholmen, J. & Halstensen, T. S. Population-based screening for celiac disease reveals that the majority of patients are undiagnosed and improve on a gluten-free diet. *Sci. Rep.* **12**, 12647 (2022). 35879335 10.1038/s41598-022-16705-2 PMC9314380

[8] Fuchs, V. et al. Delayed celiac disease diagnosis predisposes to reduced quality of life and incremental use of health care services and medicines: a prospective nationwide study. *United Eur. Gastroenterol. J.* **6**, 567–575 (2018). 10.1177/2050640617751253 PMC5987279 29881612

[9] Singh, P. et al. Global prevalence of celiac disease: systematic review and meta-analysis. *Clin. Gastroenterol. Hepatol.* **16**, 823–836e2 (2018). 29551598 10.1016/j.cgh.2017.06.037

[10] King, J. A. et al. Incidence of celiac disease is increasing over time. *Am. J. Gastroenterol.* **115**, 1 (2020). 32022718 10.14309/ajg.0000000000000523

[11] Ludvigsson, J. F. et al. Increasing incidence of celiac disease in a north American population. *Am. J. Gastroenterol.* **108**, 818–824 (2013). 23511460 10.1038/ajg.2013.60 PMC3686116

[12] Vilppula A., et al. Increasing prevalence and high incidence of celiac disease in elderly people: a population-based study. *BMC Gastroenterol.* **9**, 49 (2009). 19558729 10.1186/1471-230X-9-49 PMC2711095

[13] Pinto-Sanchez et al. Society for the study of celiac disease position statement on gaps and opportunities in coeliac disease. *Nat. Revs. Gastroenterol. Hepatol.* **18**, 875–884 (2021). 34526700 10.1038/s41575-021-00511-8 PMC8441249

[14] Pelkowski, T. D. & Viera, A. J. Celiac disease: diagnosis and management. *Am. Family Phys.* **89**, 99–105 (2014). 24444577

[15] Ianiro, G. Endoscopic tools for the diagnosis and evaluation of celiac disease. *World J. Gastroenterol.* **19**, 8562 (2013). 24379573 10.3748/wjg.v19.i46.8562 PMC3870501

[16] Al-Toma, A. et al. European society for the study of coeliac disease (ESsCD) guideline for coeliac disease and other gluten-related disorders. *United Eur. Gastroenterol. J.* **7**, 583–613 (2019). 10.1177/2050640619844125 PMC6545713 31210940

[17] Bibbins-Domingo, K. et al. Screening for celiac disease. *JAMA* **317**, 1252 (2017). 28350936 10.1001/jama.2017.1462

[18] Ludvigsson, J. F. et al. Screening for celiac disease in the general population and in high-risk groups. *United Eur. Gastroenterol. J.* **3**, 106–120 (2014). 10.1177/2050640614561668 PMC4406899 25922671

[19] Richey, R., Howdle, P., Shaw, E. & Stokes, T. Recognition and assessment of coeliac disease in children and adults: Summary of NICE guidance. *BMJ* **338**, b1684–b1684 (2009). 19474030 10.1136/bmj.b1684

[20] Chou, R. et al. Screening for celiac disease. *JAMA* **317**, 1258 (2017). 28350935 10.1001/jama.2016.10395

[21] Agardh, D. et al. Clinical features of celiac disease: a prospective birth cohort. *Pediatrics* **135**, 627–634 (2015). 25733751 10.1542/peds.2014-3675 PMC4379464

[22] Goldstein, B. A., Navar, A. M., Pencina, M. J. & Ioannidis, J. P. A. Opportunities and challenges in developing risk prediction models with electronic health records data: a systematic review. *J. Am. Med. Inf. Assoc.* **24**, 198–208 (2016). 10.1093/jamia/ocw042 PMC5201180 27189013

[23] Hyland, S. L. et al. Early prediction of circulatory failure in the intensive care unit using machine learning. *Nat. Med.* **26**, 364–373 (2020). 32152583 10.1038/s41591-020-0789-4

[24] Bosch, J. et al. A machine learning approach to liver histological evaluation predicts clinically significant portal hypertension in NASH cirrhosis. *Hepatology* **74**, 3146–3160 (2021). 34333790 10.1002/hep.32087

[25] Ye, C. et al. Prediction of incident hypertension within the next year: prospective study using statewide electronic health records and machine learning. *J. Med. Internet Res.* **20**, e22 (2018). 29382633 10.2196/jmir.9268 PMC5811646

[26] Chang, W. et al. A machine-learning-based prediction method for hypertension outcomes based on medical data. *Diagnostics* **9**, 178 (2019). 31703364 10.3390/diagnostics9040178 PMC6963807

[27] Shapiro, J. et al. Evaluation of a machine learning tool for the early identification of patients with undiagnosed psoriatic arthritis – a retrospective population-based study. *J. Transl. Autoimmun.* **7**, 100207–100207 (2023). 37577138 10.1016/j.jtauto.2023.100207 PMC10412462

[28] Rigg, J. et al. Finding undiagnosed patients with hepatitis C virus: an application of machine learning to US ambulatory electronic medical records. *BMJ Health Care Inf.* **30**, e100651. (2023). 10.1136/bmjhci-2022-100651 PMC9843171 36639190

[29] Nori, V. S. et al. Machine learning models to predict onset of dementia: a label learning approach. *Alzheimer’s Dement.* **5**, 918–925 (2019). 10.1016/j.trci.2019.10.006 PMC6920083 31879701

[30] Noda, R., Ichikawa, D. & Shibagaki, Y. Machine learning-based diagnostic prediction of IgA nephropathy: model development and validation study. *Sci. Rep.* **14**, 12426 (2024). 38816457 10.1038/s41598-024-63339-7 PMC11139869

[31] Yuan, W. et al. Accelerating diagnosis of Parkinson’s disease through risk prediction. *BMC Neurol.* **21**, (2021). 10.1186/s12883-021-02226-4 PMC8130278 34006233

[32] Wang, Y. H., Nguyen, P. A., Islam, M. M., Li, Y. C. & Yang, H. C. Development of deep learning algorithm for detection of colorectal cancer in EHR data. *Stud. Health Technol. Inform.* **264**, 438–441 . 10.3233/SHTI190259 31437961

[33] Hujoel, I. A. et al. Machine learning in detection of undiagnosed celiac disease. *Clin. Gastroenterol. Hepatol.* **16**, 1354–1355e1 (2018). 29253540 10.1016/j.cgh.2017.12.022 PMC6004230

[34] Ludvigsson, J. F. et al. Use of computerized algorithm to identify individuals in need of testing for celiac disease. *J. Am. Med. Inf. Assoc.* **20**, e306–310 (2013). 10.1136/amiajnl-2013-001924 PMC3861918 23956016

[35] Gazit, S. et al. The incidence of SARS-CoV-2 reinfection in persons with naturally acquired immunity with and without subsequent receipt of a single dose of BNT162b2 vaccine. *Ann. Intern. Med.* **175**, 674–681 (2022). 35157493 10.7326/M21-4130 PMC8855786

[36] Husby, S. et al. European society for pediatric gastroenterology, hepatology, and nutrition guidelines for the diagnosis of coeliac disease. *J. Pediatr. Gastroenterol. Nutr.* **54**, 136–160 (2012). 22197856 10.1097/MPG.0b013e31821a23d0

[37] Ciacci, C. et al. Serum anti-tissue transglutaminase IgA and prediction of duodenal villous atrophy in adults with suspected coeliac disease without IgA deficiency (Bi.A.CeD): a multicentre, prospective cohort study. *Lancet Gastroenterol. Hepatol.* **8**, 1005–1014 (2023). 37696284 10.1016/S2468-1253(23)00205-4

[38] Werkstetter, K. J. et al. Accuracy in diagnosis of celiac disease without biopsies in clinical practice. *Gastroenterology* **153**, 924–935 (2017). 28624578 10.1053/j.gastro.2017.06.002

[39] Piccialli, F. et al. Precision medicine and machine learning towards the prediction of the outcome of potential celiac disease. *Sci. Rep.* **11**, (2021). 10.1038/s41598-021-84951-x PMC7952550 33707543

[40] Lee, S., Lee, H., Choi, J. R. & Koh, S. B. Development and validation of prediction model for risk reduction of metabolic syndrome by body weight control: a prospective population-based study. *Sci. Rep.* **10**, (2020). 10.1038/s41598-020-67238-5 PMC7305222 32561810

[41] Shwartz-Ziv, R. & Armon, A. Tabular data: deep learning is not all you need. *Inf. Fusion* **81**, 84–90 (2022).

[42] Pedregosa, F. et al. Scikit-learn: machine learning in python. *J. Mach. Learn. Res.* **12**, 2825–2830 (2011).

[43] Jessup, A. B., Law, J. R. & Spagnoli, A. Are HDL levels lower in children with type 1 diabetes and concurrent celiac disease compared with children with type 1 diabetes only? *J. Pediatr. Endocrinol. Metab.* **27**, 1213–1216 (2014). 25153213 10.1515/jpem-2013-0464

[44] Lundberg, S. & Lee, S. I. A unified approach to interpreting model predictions. *arXiv preprint arXiv:1705.07874* (2017).

[45] Sainsbury, A., Sanders, D. S. & Ford, A. C. Meta-analysis: Coeliac disease and hypertransaminasaemia. *Aliment. Pharmacol. Ther.* **34**, 33–40 (2011). 21545472 10.1111/j.1365-2036.2011.04685.x

[46] Volta, U. Pathogenesis and clinical significance of liver injury in celiac disease. *Clin. Rev. Allergy Immunol.* **36**, 62–70 (2008). 10.1007/s12016-008-8086-x 18496773

[47] Salardi, S. et al. Whole lipid profile and not only HDL cholesterol is impaired in children with coexisting type 1 diabetes and untreated celiac disease. *Acta Diabetol.* **54**, 889–894 (2017). 28639064 10.1007/s00592-017-1019-5

[48] Abu Daya, H., Lebwohl, B., Smukalla, S., Lewis, S. K. & Green, P. H. Utilizing HDL levels to improve detection of celiac disease in patients with iron deficiency anemia. *Am. J. Gastroenterol.* **109**, 769–770 (2014). 24797006 10.1038/ajg.2014.30

[49] Bergamaschi, G. et al. Anemia of chronic disease and defective erythropoietin production in patients with celiac disease. *Haematologica* **93**, 1785–1791 (2008). 18815191 10.3324/haematol.13255

[50] Jena, A. et al. Liver abnormalities in celiac disease and response to gluten-free diet: a systematic review and meta-analysis. *J. Gastroenterol. Hepatol.* **38**, 11–22 (2022). 36300634 10.1111/jgh.16039

[51] Brar, P. et al. Change in lipid profile in celiac disease: beneficial effect of gluten-free diet. *Am. J. Med.* **119**, 786–790 (2006). 16945614 10.1016/j.amjmed.2005.12.025

[52] Nikniaz, Z., Farhangi, M. A., Hosseinifard, H. & Nikniaz, L. Does a gluten-free diet increase body mass index and lipid profile in celiac patients? A systematic review and meta-analysis. *Mediterr. J. Nutr. Metab.* **12**, 341–352 (2019).

[53] Kurppa, K. et al. Benefits of a gluten-free diet for asymptomatic patients with serologic markers of celiac disease. *Gastroenterology* **147**, 610–617e1 (2014). 24837306 10.1053/j.gastro.2014.05.003

[54] Vilppula, A. et al. Clinical benefit of gluten-free diet in screen-detected older celiac disease patients. *BMC Gastroenterol.* **11**, (2011). 10.1186/1471-230X-11-136 PMC3377922 22176557

[55] Galli, G. et al. Relationship between persistent gastrointestinal symptoms and duodenal histological findings after adequate gluten-free diet: a gray area of celiac disease management in adult patients. *Nutrients* **13**, 600 (2021). 33673062 10.3390/nu13020600 PMC7918091

[56] Patel, N. et al. Clinical data do not reliably predict duodenal histology at follow-up in celiac disease. *Am. J. Surg. Pathol.* **48**, 212–220 (2023). 37994653 10.1097/PAS.0000000000002150

[57] Norström, F., Lindholm, L., Sandström, O., Nordyke, K. & Ivarsson, A. Delay to celiac disease diagnosis and its implications for health-related quality of life. *BMC Gastroenterol.* **11**, (2011). 10.1186/1471-230X-11-118 PMC3233515 22060243

[58] Mårild, K. et al. Costs and use of health care in patients with celiac disease: a population-based longitudinal study. *Am. J. Gastroenterol.* **115**, 1253–1263 (2020). 32349030 10.14309/ajg.0000000000000652

[59] Paavola, S. et al. Coeliac disease re-screening among once seronegative at‐risk relatives: a long‐term follow‐up study. *United Eur. Gastroenterol. J.* **10**, 585–593 (2022). 10.1002/ueg2.12255 PMC9278577 35611878

[60] Rubio-Tapia, A. et al. American college of gastroenterology guidelines update: diagnosis and management of celiac disease. *Am. J. Gastroenterol.* **118**, 59–76 (2023). 36602836 10.14309/ajg.0000000000002075

