# Supplementary material for: A machine learning tool for early identification of celiac disease autoimmunity
Source: Sci Rep. 2024 Dec 28;14:30760. doi: 10.1038/s41598-024-80817-0 (PMC11681168; doi:10.1038/s41598-024-80817-0)
Supplement: Supplementary file 1 — Supplementary Material 1 [file 41598_2024_80817_MOESM1_ESM.docx]

| **Candidate features** |
| --- |
| Age |
| Biological Sex |
| Basophils (n) |
| Basophils (%) |
| Eosinophils (n) |
| Eosinophils (%) |
| Hematocrit |
| Hemoglobin |
| Lymphocytes (n) |
| Lymphocytes (%) |
| MCH |
| MCHC |
| MCV |
| Monocytes (n) |
| Monocytes (%) |
| MPV |
| Neutrophils (n) |
| Neutrophils (%) |
| Platelets |
| RBC |
| RDW |
| WBC |
| Albumin |
| ALKP |
| ALT |
| AST |
| Bilirubin Total |
| Calcium |
| Creatinine |
| Glucose |
| Potassium |
| Sodium |
| Phosphor |
| Protein Total |
| Urea |
| Uric Acid |
| Ferritin |
| HDL |

**Supplementary Table S1.** Laboratory and demographic features used as model input.

| **Algorithm** | **Package** | **Tuning Parameters** |
| --- | --- | --- |
| Logistic regression | LogisticRegression | C, penalty |
| Decision tree | DecisionTreeClassifier | max_depth, criterion |
| Random forest | RandomForestClassifier | n_estimators, max_depth |
| XGBoost | GradientBoostingClassifier | learning_rate, n_estimators, max_depth, reg_alpha, min_child_weight, max_bin |
| Multilayer perceptron | MLPClassifier | learning_rate_init, hidden_layer_sizes |

**Supplemental Table S2**. Tuning parameters used for each machine learning algorithm.

| **Model** | **Gap** | | | |
| --- | --- | --- | --- | --- |
|  | 1-year | 2-year | 3-year | 4-year |
| XGBoost | 85.81 | 82.94 | 82.46 | 81.38 |
| LR | 84.55 | 82.71 | 81.42 | 81.00 |
| RF | 82.76 | 79.69 | 78.56 | 78.13 |
| MLP | 80.46 | 79.53 | 77.54 | 74.59 |
| DT | 77.14 | 72.12 | 71.15 | 70.;23 |

**Supplementary Table S3.** AUC of each model as measured in the highly seropositive population (tTG-IgA>10X ULN) vs controls. The models tested are XGBoost, logistic regression (LR), random forest (RF), multilayer perceptron (MLP) and decision tree (DT).

| **Model** | **Gap** | | | |
| --- | --- | --- | --- | --- |
|  | 1-year | 2-year | 3-year | 4-year |
| XGBoost | 79.09 | 76.70 | 74.75 | 75.25 |
| LR | 77.71 | 76.37 | 75.57 | 74.39 |
| RF | 78.38 | 75.08 | 74.05 | 74.28 |
| MLP | 74.79 | 72.45 | 70.51 | 69.37 |
| DT | 72.10 | 68.56 | 68.31 | 68.42 |

**Supplementary Table S4.** AUC of each model as measured in the seropositive population (tTG-IgA>2X ULN) vs controls. The models tested are XGBoost, logistic regression (LR), random forest (RF), multilayer perceptron (MLP) and decision tree (DT).
